# Small-Group Teaching: Should It Be Recorded?

**DOI:** 10.1007/s40670-023-01837-5

**Published:** 2023-07-25

**Authors:** Peter Crook, Shagayegh Javadzadeh, Rebecca Shone, Vikram Joseph, Debasish Banerjee, Nicholas M. P. Annear

**Affiliations:** 1 Institute of Medical and Biomedical Education, St George’s, University of London, Cranmer Terrace, London, SW17 0RE UK; 2 Department of Acute Medicine, St George’s Hospital, St George’s University Hospitals NHS Foundation Trust, Blackshaw Road, London, SW17 0QT UK; 3 Department of Renal Medicine, St George’s Hospital, St George’s University Hospitals NHS Foundation Trust, Blackshaw Road, London, SW17 0QT UK

**Keywords:** Medical education, Small-group learning, Case-based learning, Recording

## Abstract

**Background:**

Recording large-group lectures is commonplace in higher education, allowing students to access content asynchronously and remotely. With the move towards online learning during the COVID-19 pandemic, recording of small-group teaching sessions has also become increasingly common; however, the educational value of this practice is unknown.

**Methods:**

All medical students rotating through the Acute Medicine Department of a large teaching hospital were invited to enrol in the study. Consenting students were recorded for the second half of an online case-based learning (CBL) session. The recording was available for 6 months; viewing patterns were analysed. Students were sent a questionnaire after the session, asking them to reflect on the recorded and unrecorded halves of the session.

**Findings:**

Thirty-three students underwent recording in 12 separate groups; 31 students (94%) completed the questionnaire. All 31 respondents (100%) described the session as “useful” or “very useful”. Twenty-four respondents (77%) recommended continuing to record small-group sessions and 17 (55%) reported being “likely” or “very likely” to watch the recording. Six respondents (19%) reported a negative impact of being recorded. During 6 months of follow-up, no students returned to view the recording for more than 1 minute.

**Conclusion:**

Despite positive feedback for the session and high student demand for ongoing recording, no students viewed the recording for any significant duration. One-fifth of students reported a negative impact of being recorded. The findings from this study do not support routine recording of small-group CBL sessions, even where demand for this may exist.

**Supplementary Information:**

The online version contains supplementary material available at 10.1007/s40670-023-01837-5.

## Background

The COVID-19 pandemic saw a dramatic increase in the use of online platforms for medical education. Online learning facilitated training in an era where social distancing requirements rendered the classroom and lecture theatre virtually obsolete [1–3]. Even now that social distancing requirements have eased, a significant proportion of medical education remains online, taking advantage of the ability to learn and teach remotely, either from home or whilst on clinical rotation. Online learning has been shown to improve attendance and a majority of students support online learning playing a more prominent role in post-pandemic medical education [1].

The paradigm shift towards online learning has brought a new wave of recorded video content, as students increasingly indicate a preference for electronic resources that they can review asynchronously [1]. Moreover, recording teaching sessions has become effortless, as video-conferencing software allows seamless recording of online sessions without any additional logistic or hardware requirements.

Recording of large-group lectures (known as lecture capture) is now commonplace in higher education institutions, with over 80% of UK institutions facilitating this practice [4, 5]. There are abundant resources to support institutions in utilising lecture capture to create a more inclusive learning environment [6–8]. In contrast, little is known about the educational value of recording small-group sessions. Students are overwhelmed with a choice of online resources and whether they have the time or inclination to re-watch small-group sessions is unclear. This is particularly true of case-based learning (CBL) sessions, where the principal goal is not information acquisition but development of critical thinking and collaborative skills. The decision to record such sessions should be considered carefully as the capture and storage of these recordings has a range of potential implications, including ethical, welfare, financial, and environmental concerns [9, 10].

This study was devised in response to repeated requests to record an online CBL session. We sought to evaluate whether students supported the recording of small-group teaching sessions delivered online and to determine whether these recordings were of any educational value.

## Methods

This prospective, single-centre cohort study was undertaken in the Acute Medicine Department of a large inner-city teaching hospital in London, UK, between April and July 2021. Study participants were medical students in their penultimate or final year of study, rotating through the department for a 1-week clinical placement. Students at that time were receiving a blend of online and in-person teaching.

All medical students rotating through the department during the study period were invited to enrol in the study. They were emailed the Participant Information Sheet (PIS; Supplementary Data 1) during the week prior to their rotation and were offered the chance to ask questions during their induction. Participants were informed that enrolment was voluntary and would not affect the quantity or quality of teaching received, nor their placement sign-off. Students who wished to enrol provided informed, written consent.

The study involved recording of an online, case-based learning session that already formed an integral part of the students’ 1-week placement. The session, called the “Virtual Ward Round”, took the form of a 3-hour case-based learning session involving 2–5 students. It was hosted online via Microsoft Teams® (Microsoft Corporation, WA, USA) and was facilitated by one of the study investigators in their capacity as a Clinical Teaching Fellow (CTF). Students would take turns in leading their colleagues through one of six fictional clinical cases. They would be encouraged to take ownership of the case and to talk through their interpretation and management in collaboration with their colleagues.

The session itself was already a mandatory part of the students’ rotation; recording of the session through participation in the study was, however, entirely voluntary. Provided all participating students consented to study enrolment, the facilitator recorded the second half of the session using Microsoft Teams®’ built-in recording function. The students were recorded for the second half only so that they could serve as self-controls, reflecting on a period of both recorded and non-recorded teaching. The nature of the session, which comprised of six discrete clinical cases, allowed recording of only half of the session without compromising the value of the recorded half. This recording was then hosted securely on a Video Content Management System (Panopto, Inc., Seattle, USA), with data storage costs met by the University.

Students were emailed a link through which they could access their group’s recording for a period of 6 months after the session. The Video Content Management System allowed students to re-watch the session at their convenience, including the ability to alter the viewing speed and pause, skip, and re-watch particular sections. The system automatically logged the frequency and duration of views. These data were available to the study investigators only. Immediately following the session, students were also sent an online questionnaire (Supplementary Data 2), asking them to reflect on their satisfaction with the session, the experience of being recorded, and self-reported likelihood of returning to watch the recording.

Data analyses were performed in R version 4.2.1 (R Foundation for Statistical Computing) using additional libraries: ggplot, gtsummary, HH, and tidyverse. Categorical data are presented as number (percentage). Differences in Likert responses for the two halves of the session (recorded and unrecorded) were assessed as paired, non-parametric data, using a two-sided Wilcoxon signed rank test. Qualitative responses were assessed manually to identify themes.

This study received approval from the Research and Ethics Committee of St George’s, University of London (2021.0085).

## Findings

### Study Cohort

All 37 students (100%) rotating through the department during the study period consented to enrolment in the study. There was a recording failure for one group, meaning that a total of 33 students, in 12 separate groups, were included for study analysis. Sixteen identified as female, 14 male, and 3 did not respond (see Table 1). Twenty-three students were on the standard 5-year undergraduate MBBS programme; seven students were on the graduate-entry, 4-year MBBS programme [3 did not respond]. The mean group size was 2.75 students (range 2 to 4).

**Table 1:** Demographics of cohort. Categorical variables are given as number (% of those who responded); age is presented in years as mean (range)

**Cohort characteristics**	***N*** **= 33**
**Gender**	
Female Male * Did not respond*	16 (53%) 14 (47%) *3*
**Age**	23 (20–28)
*Did not respond*	*11*
**Ethnicity**	
White Asian or Asian British Black or Black British Mixed Other * Did not respond*	14 (47%) 12 (40%) 2 (7%) 1 (3%) 1 (3%) *3*
**Medical course**	
5-year MBBS 4-year MBBS (*graduate entry*) * Did not respond*	23 (77%) 7 (23%) *3*

### Feedback on the Session

Thirty-one out of 33 students (94%) completed the online questionnaire. All 31 respondents (100%) felt that the session was “very useful” or “useful” and “very relevant” or “relevant” to their learning needs. Twenty-nine respondents (94%) agreed or strongly agreed with the statement, “I felt engaged even when it was not my turn to present”; no respondents disagreed with this statement. When asked how the session compared to a traditional ward round, 30 respondents (97%) gave a positive response, with 21 (68%) explicitly suggesting that it was better than a traditional ward round. All 31 respondents (100%) advocated for continuing this session; three (10%) suggested that it would be better if delivered face-to-face rather than online.

### Feedback on Recording

**Fig. 1:**
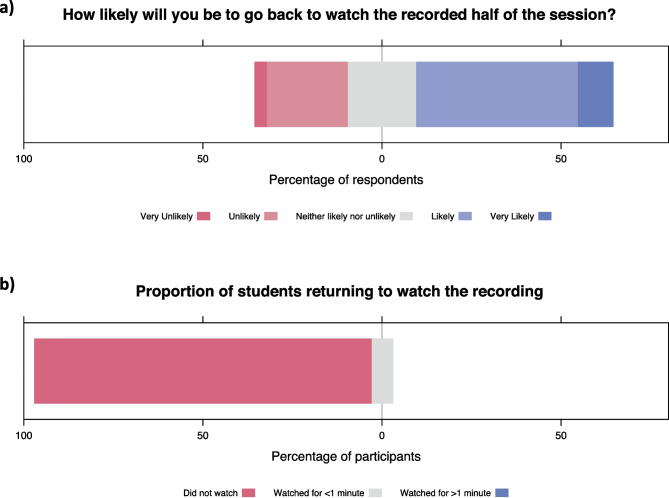
**a** Likert responses to the question: “How likely will you be to go back to watch the recorded half of the session?”. **b** Recording data from the Video Content Management System showing proportion of students who watched the recording during 6 months of follow-up

Twenty-four respondents (77%) recommended continuing to record small-group sessions such as this. Seventeen (55%) suggested that they would be “very likely” or “likely” to go back and watch the recording of the session in future (see Fig. 1a).

Reasons given for not supporting recording of the session included that the presentation slides alone would be sufficient to revise the material (5 respondents [16%]) and that re-watching the recording would be too time-consuming (5 respondents [16%]). One respondent (3%) mentioned that recording “would probably increase anxiety toward the session slightly” and another that “participating and thinking on the spot was more beneficial [than re-watching the recording]”. In contrast, nineteen respondents (61%) did specifically reference a benefit or utility in returning to re-watch the recording. Specific reasons given included references to use of the recording to support revision (5 respondents [16%]); a benefit from not needing to take notes (2 respondents [6%]); and being able to recap missed points (2 respondents [6%]).

When asked about the impact of recording on the session, 25 out of 31 respondents (81%) denied any impact; 6 out of 31 (19%), however, did report an impact. Two respondents (6%) mentioned choosing not to put their camera on; 4 (13%) mentioned feeling “self-conscious”, “daunted”, or “thinking about answers more”.

**Fig. 2:**
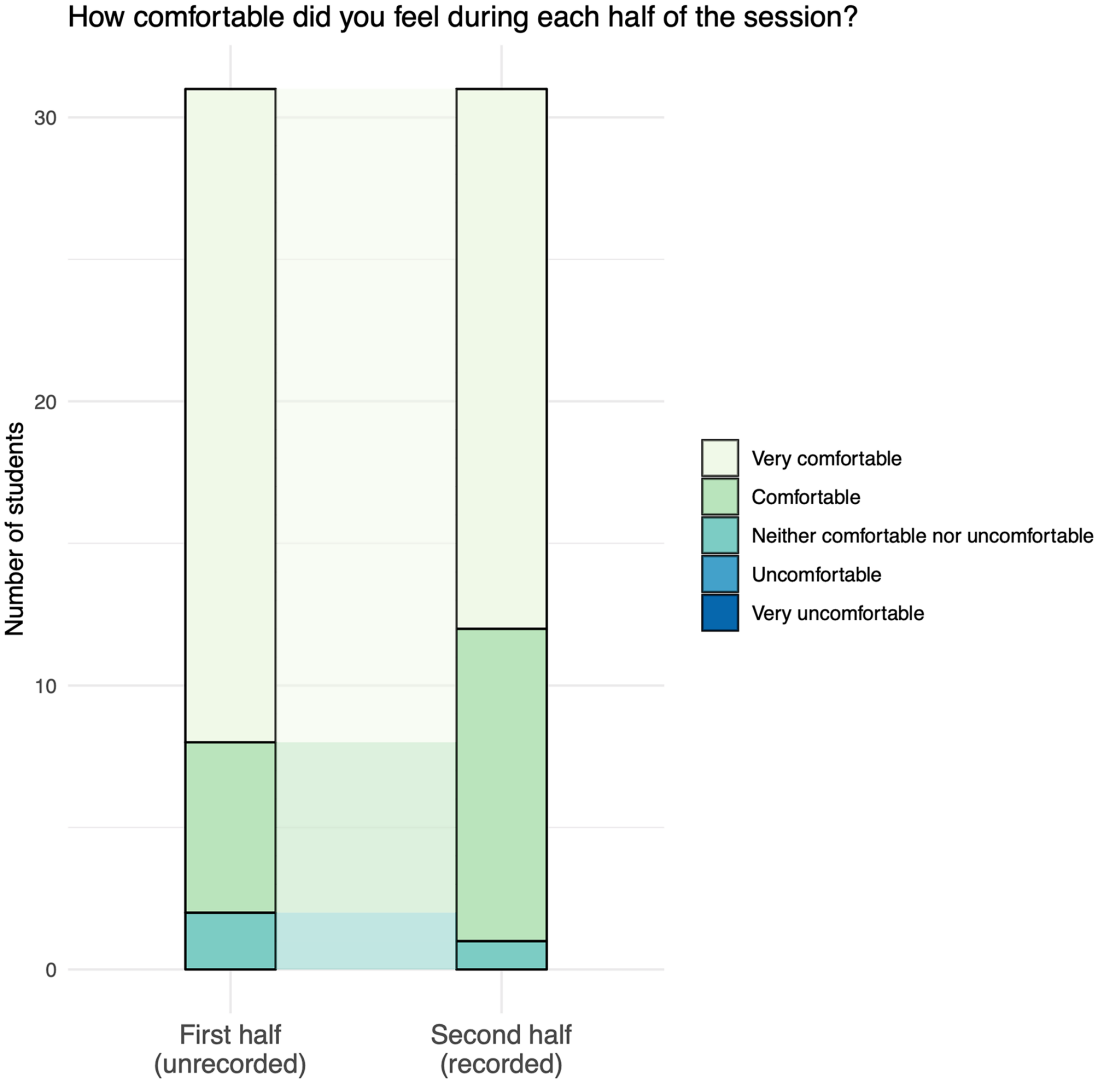
Graph showing reported comfort during the unrecorded (first) and recorded (second) halves of the session

Participants were asked to reflect on their comfort during both parts of the session (non-recorded and recorded). Twenty-nine respondents (94%) reported feeling “comfortable” or “very comfortable” for both parts; no respondents reported being “uncomfortable” or “very uncomfortable” at any point. Four respondents (13%) downgraded from feeling “very comfortable” to “comfortable” for the recorded part of the session; one respondent (3%) upgraded from feeling “neither comfortable nor uncomfortable” to “comfortable” for the second half. There was no significant difference in comfort between the two halves (*p* = 0.23; see Fig. 2).

### Recording Data

During the 6-month follow-up period, 2 students (6%) returned to view the recording, both watching for 1 min only. Both views occurred within 2 weeks of the link being sent. The remaining 31 students (94%) never opened the recording (see Fig. 1b).

The mean recording duration was 75 min (range 53 to 100 min).

## Discussion

In this cohort from a medical school in London, UK, there was a significant discrepancy between student demand for recording of a small-group case-based learning (CBL) session and subsequent viewing practices. The CBL session received uniformly positive feedback, and a significant majority of students suggested that they would return to view the recording; however, in the 6-month follow-up period, no student returned to view the recording for a meaningful duration. Concerningly, almost one-fifth of students reported a negative impact from being recorded, specifically in terms of feeling more self-conscious or electing to turn their camera off.

The recording of large-group lectures (known as lecture capture) has become common practice in higher education establishments [4] and is valued by students [11–13]. Similarly, use of video recording to facilitate feedback in communication skills teaching has significant benefits as an aid for self-reflective practice [14]. Recording of small-group CBL sessions, however, is less common and, to our knowledge, this is the first study to evaluate this practice. In the absence of specific guidance on this topic, this study was designed to inform the response to repeated student requests to record online CBL sessions.

Despite high levels of student demand for recording, both before and during the study, this demand was not reflected in subsequent viewing practices. The reasons for this discrepancy are unknown. The proven value of lecture capture may be restricted to those students who miss the original lecture and watch it for the first time via the recording. This subgroup of students, however, were not included in this study: absent students were intentionally not offered access to the recording in case this discourage engagement and interaction from those present in the session. Moreover, the value of re-watching a didactic, content-based lecture may differ from the value added by re-watching a CBL session; for lectures, the reiteration of key points may help to consolidate knowledge, whilst there may be limited value in repeating the procedural learning of a CBL session.

CBL is a widely used approach in medical education that enables students to apply their knowledge to real-world scenarios, promoting higher levels of cognition through guided inquiry. It is constructivist by nature: students form new meanings by interacting with their knowledge and the environment [15]. Williams (2005) describes CBL as a collaborative process that facilitates the integration of learning. The use of authentic cases promotes students’ intrinsic and extrinsic motivation to learn, encourages self-reflective practice, and allows them to integrate their knowledge and skills through scientific inquiry [16]. Learner engagement is thus a prerequisite for effective learning through CBL and collaborative interaction is central to its value as an educational event; the absence of these elements in passively re-watching a recording likely limits the value of recording such sessions.

The decision to record individual teaching sessions needs careful consideration, as the recording, curating, and storing of teaching sessions is associated with significant costs. In addition to the negative impact on student engagement reported in this study, there is a financial and environmental cost to the online storage of data. Based on a predicted file size of 400 megabytes per hour [17], recording of each of these sessions would average 500 megabytes of online storage. Sustaining this practice of keeping recordings for 6 months would require 13 gigabytes of storage on an online management system for one CBL session alone. Supporting this practice across a university programme — or indeed scaling up across the whole sector — would incur far more significant costs. The academic discourse surrounding the impact of online education on an institution’s carbon footprint continues to develop [18]. There are carbon emissions associated with online data storage, in large part due to the cooling requirements for servers in large data centres [19]. There is also a potential financial implication, with Video Content Management Systems charging institutions for data storage, and an increased administrative burden for university staff. Finally, the storage of students’ personal data and video presents a confidentiality risk if systems are compromised or if students breach their institution’s code of conduct around online material.

This study had several limitations, including a small cohort size and the use of a single teaching session as the substrate for recording. Although feedback was anonymous, the results could be identified at group-level, and this may have influenced responses. Finally, the study was conducted towards the end of the academic year, and this may also have impacted viewing practices. Whilst further research is needed to generalise these results to other settings, the findings of this study should encourage educators to consider carefully the potential benefits and harms of recording small-group sessions, prior to agreeing to individual student requests to do so. This is particularly important for CBL sessions where the act of recording may itself negatively impact upon the value of the learning session to those present.

## Conclusion

Online learning has been adopted as an integral part of medical education, even after the lifting of social distancing requirements. This study has shown no benefit to recording a small-group online CBL session and has found a potentially negative impact on student engagement. These findings, together with the potential financial and environmental impacts of recording, should give educators pause for thought prior to recording small-group CBL sessions, even where student demand for this may exist.

### Supplementary Information

Below is the link to the electronic supplementary material.

Supplementary file1 (PDF 78 KB)

Supplementary file2 (PDF 77 KB)

## Data Availability

Not applicable.
