# Supplementary material for: Small-Group Teaching: Should It Be Recorded?
Source: Med Sci Educ. 2023 Jul 25;33(5):1073–9. doi: 10.1007/s40670-023-01837-5 (PMC10597944; doi:10.1007/s40670-023-01837-5)
Supplement: Supplementary file 1 — Supplementary file1 (PDF 78 KB) [file 40670_2023_1837_MOESM1_ESM.pdf]

# Participant Information Sheet (PIS)

**Study title:** Recording small group teaching sessions: what is the educational value?

**REC Reference Number: 2021.0085**

**Participants will be given a copy of this information sheet**

As the Clinical Teaching Fellows for Acute Medicine at St George's University Hospitals NHS Foundation Trust, we would like to invite you to participate in a research project that we are conducting about the educational value of recording small group teaching sessions.

You should only participate if you want to; choosing not to take part will not disadvantage you in any way and will not affect the teaching delivered. Before you decide whether you want to take part, it is important for you to understand why the research is being done and what your participation will involve. Please take time to read the following information carefully and discuss it with others if you wish. Please feel free to ask us if there is anything that is not clear or if you would like more information.

This study has been given a favourable ethical opinion by the St George`s Research Ethics Committee.

***What is the purpose of the study?***

The aim of this study is to identify whether there is any value in recording small group teaching sessions that take place online. As you may know, it is common practice to record large group lectures; for small group sessions, practice varies and we would like to know whether we should be recording these as well.

We are specifically interested in knowing whether students are likely to go back to watch small group sessions if they are recorded, but also to know whether the recording of these sessions impacts on your willingness to engage and interact.

***Why have I been invited to take part?***

We are inviting all SGUL students doing their one-week P-year rotation on Acute Medicine at St George's Hospital.

We are aiming to recruit participants for 10 sessions (approx. 40 to 50 students in total) so that we have good representation of your cohort.

***Do I have to take part?***

No, participation is voluntary. You do not have to take part. You should read this information sheet and if you have any questions you should ask the research team.

***What will happen to me if I take part?***

As part of your teaching on AMU, we run a Virtual Ward Round on Microsoft Teams on Wednesday mornings. This session will take place irrespective of whether you agree to be

part of the study as it is a standard part of the week. However, we do not routinely record this session.

If all students from your group consent, we will record the second half of the session (both audio and video recording via Teams®, including the facilitator's slideshow). The recording will be stored on Panopto with access granted ***only*** to those students who were present during the session and to the three Clinical Teaching Fellows on AMU. You will be free to go back to watch the recording at any time if you find it useful, although there is no obligation to do so.

If all students consent, we will also ask you to fill out a brief survey about the teaching session, including routine questions about how you found the session, but also some questions about whether you were conscious of being recorded and whether this impacted on your learning.

The recording will be visible to you and your colleagues in your group only and will be deleted after 6 months. We will monitor how many times the video has been viewed using routine analytics captured by Panopto (number of views and duration of views). All data will be fully anonymised at the point of analysis. Your recordings will **not** be published or circulated outside of the students in your session and the three Clinical Teaching Fellows for Acute Medicine.

We will aim to publish the anonymised survey responses and Panopto data so that we can assess whether or not there is any value in recording small group sessions.

If you decide to take part you will be given this information sheet to keep and will be asked to sign a consent form.

You will be able to withdraw consent at any time and the recording will be removed from Panopto and deleted.

As part of your written consent, you will be asked to confirm that you will not download, circulate or share the recording yourself.

## ***What are the possible benefits and risks of taking part?***

The information from this study will help to inform practice at SGUL and in other higher education settings, where there is uncertainty about whether or not to record small group tutorials. By taking part you will be helping to inform our practice and also potentially the practice of other higher education institutions. The results may be generalisable to a wide range of disciplines.

A possible disadvantage to taking part in the study is that some students may not feel comfortable being recorded and may feel less inclined to engage.

The project has no outside funding.

## ***What if something goes wrong?***

If you wish to make a complaint about the conduct of the study you can contact using the details below for further advice and information:

Dr Nicholas Annear: Academic Lead for P years, Lead for Medicine Clinical Placements
nannear@sgul.ac.uk

The Trust has in force the relevant insurance policies which apply to this study. If you wish to complain, or have any concerns about any aspect of the way you have been treated during the course of this study then you should follow the instructions given above.

## ***Who should I contact for further information?***

If you have any questions or require more information about this study, please contact one of the Clinical Teaching Fellows using the following contact details:

Dr Peter Crook: pcrook@sgul.ac.uk
Dr Rebecca Shone: rshone@sgul.ac.uk
Dr Vikram Joseph: vjoseph@sgul.ac.uk

All at: Department for Acute Medicine, Richmond Ward, St James Wing, St George's Hospital, Blackshaw Rd, Tooting, London SW17 0QT

## ***What data will be collected? How and where data will be stored? How long data will be kept?***

As a publicly-funded organisation, we have to ensure when we use identifiable personal information from people who have agreed to take part in research, this data is processed fairly and lawfully and is done so on the basis of **public interest**. This means that when you agree to take part in this research study, we will use your data in the ways needed to conduct and analyse the research study.

Your feedback responses will be anonymous – we will not ask for your name or student number on the response form. The responses will be downloaded and stored within a secure folder on the NHS server for St George's University Hospitals NHS Foundation Trust. The file will be encrypted and password-protected with access only granted to the three Clinical Teaching Fellows for Acute Medicine.

Panopto routinely collects data on who views videos, including the name, the number of views and the duration of views. This will be stored within Panopto. All data regarding viewing practices will be anonymised prior to analysis. Analysis will be performed by the three Clinical Teaching Fellows for Acute Medicine. Access to the video recordings will only be granted to the students present in the session and the three CTFs for Acute Medicine.

The consent forms, Panopto viewing data and survey responses will be kept for 5 years (according to SGUL data management regulation). The recording itself, however, will be deleted after 6 months.

## ***Who is Handling My Data?***

St George's University of London as the sponsor, will act as the 'Data Controller' for this study. We will process your personal data on behalf of the controller and are responsible for looking after your information and using it properly. This information will include your anonymised feedback responses and the routine analytics about your viewing of the recording on Panopto (number and duration of views by each user). We will use this information as explained in the 'What is the purpose of the study' section above.

## ***What happens if I change my mind?***

You have the right to change your mind and withdraw from the study without giving a reason. You will be able to withdraw consent at any time during the 6 months and the recording will be removed from Panopto and deleted. You will not be able to withdraw your survey response, however, as it will be anonymised.

You can find out more about how we use your information:
**https://www.sgul.ac.uk/privacy**

Or contact our university Data Protection Officer at:
**Email:** **dataprotection@sgul.ac.uk**
**Tel:** **020 8725 0668**

## ***What will happen to the results of the study?***

We aim to publish the results of this study in a peer-reviewed journal. The results may also be presented in a poster format at a conference. All data will be fully anonymised at the point of analysis and publication. The video recordings themselves will not feature in any publication or any part of the analysis.

## ***Will my data be used for future research?***

The anonymised results of the study will be published. The Panopto data will only be used for this study and will not form part of any other research. The survey responses may be used in other publications regarding the use of Virtual Ward Round as a teaching modality. These survey responses are fully anonymised.

## ***What if I want to complain about the way data is handled?***

If you wish to raise a complaint on how we have handled your personal data, you can contact our Data Protection Officer who will investigate the matter. If you are not satisfied with our response or believe we are processing your personal data in a way that is not lawful you can complain to the Information Commissioner's Office (ICO) (https://ico.org.uk/).

## ***Data Protection Privacy Notice***
St George`s University London conducts research to the highest standards of research integrity. As a publicly-funded organisation, the University has to ensure that it is in the public interest when we use personally-identifiable information about people who have agreed to take part in research. The University's data protection policy governing the use of personal

data by the University can be found on its website (https://www.sgul.ac.uk/about/our-professional-services/information-services/information-governance/data-protection/data-protection-policy).

Any personal data we collect in this study will be used only for the purposes of carrying out our research and will be handled according to the University's policies in line with data protection law. If any personal data is used from which you can be identified directly, it will not be disclosed to anyone else without your consent unless the St George London University is required by law to disclose it.
For the purposes of data protection law, the University is the 'Data Controller' for this study, which means that we are responsible for looking after your information and using it properly. It will keep identifiable information about you for 5 years after the study has finished after which time any link between you and your information will be removed.

**Thank you for reading this information sheet and for considering taking part in this research.**
