# Supplementary material for: Small-Group Teaching: Should It Be Recorded?
Source: Med Sci Educ. 2023 Jul 25;33(5):1073–9. doi: 10.1007/s40670-023-01837-5 (PMC10597944; doi:10.1007/s40670-023-01837-5)
Supplement: Supplementary file 2 — Supplementary file2 (PDF 77 KB) [file 40670_2023_1837_MOESM2_ESM.pdf]

Recording small group teaching sessions - Survey Form

REC Reference Number: 2021.0085

Thank you for agreeing to complete this feedback survey about the AMU Virtual Ward Round and your experience of being recorded. Completing this form remains optional; choosing not to complete it will not disadvantage you in any way.

This survey has been given a favourable ethical opinion by the St George`s Research Ethics Committee.

The survey is anonymous – please do not submit your name or student number anywhere on the form.

The Questionnaire has 17 questions and should take approximately 5 minutes to complete.

Responses to this survey will be downloaded onto a secure, password-protected folder on the NHS Trust server and will be retained for 5 years.

Participation is completely voluntary, and you can stop at any time. Please note, however, that anonymous data (such as this questionnaire) cannot be withdrawn after it has been submitted.

Where can I get more information?
If you have any questions or concerns, please contact the Clinical Teaching Fellows (Peter Crook: pcrook@sgul.ac.uk (mailto:pcrook@sgul.ac.uk); Rebecca Shone: rshone@sgul.ac.uk (mailto:rshone@sgul.ac.uk); Vikram Joseph: vjoseph@sgul.ac.uk (mailto:vjoseph@sgul.ac.uk)).

The online survey will be available for 3 months after your session, although please submit it as soon as possible and only once.

Thank you for reading this information section and for taking part in this research.

* Required

1. Check this box to indicate you have read and understood the information section, and that you consent to participate in this survey *

○

Demographics

2. What is your age? [optional]

3. What is your gender?

- Female
- Male
- Non-binary
- Prefer not to say

4. What is your ethnicity?

- White
- Mixed
- Asian or Asian British
- Black or Black British
- Other
- Prefer not to say

5. Which programme are you on?

- ◯ MBBS 5
- ◯ MBBS 4
- ◯ International MBBS
- ◯ Prefer not to say

Feedback on recording

6. Did the recording of the second half of the session impact you in any way? *

7. During the first half of the session (not recorded), how comfortable did you feel participating in the session? *

- Very comfortable
- Comfortable
- Neither comfortable nor uncomfortable
- Uncomfortable
- Very uncomfortable

8. During the second half of the session (recorded), how comfortable did you feel participating in the session? *

- Very comfortable
- Comfortable
- Neither comfortable nor uncomfortable
- Uncomfortable
- Very uncomfortable

9. Would you recommend continuing to record this small group teaching session and why / why not? *

Feedback on the session

10. In terms of supporting your learning, how did Virtual Ward Round compare to a traditional ward round? In what ways was it better; in what ways was it worse? *

11. Would you advocate continuing this session for your future colleagues? Or would you remove it / alter it, and if so how? *

12. How relevant was this session to your learning needs? *

- ○ Very relevant
- ○ Relevant
- ○ Neither relevant nor irrelevant
- ○ Not relevant
- ○ Not at all relevant

13. How useful did you find this session? *

- ◯ Very useful
- ◯ Useful
- ◯ Neutral
- ◯ Not useful
- ◯ Not at all useful

14. The pace of the session was: *

- ◯ Too fast
- ◯ About right
- ◯ Too slow

15. I felt engaged even when it was not my turn to present *

- ◯ Strongly agree
- ◯ Agree
- ◯ Neither agree nor disagree
- ◯ Disagree
- ◯ Strongly disagree

16. How likely will you be to go back to watch the recorded half of the session *

- Very likely
- Likely
- Neither likely nor unlikely
- Unlikely
- Very unlikely

17. How have you found the process of being involved in educational research?

11/19/2021

This content is neither created nor endorsed by Microsoft. The data you submit will be sent to the form owner.

Microsoft Forms
